# Technology and Universal Health Coverage: Examining the role of digital health

**DOI:** 10.7189/jogh.11.16006

**Published:** 2021-11-20

**Authors:** David Wilson, Aziz Sheikh, Marelize Görgens, Katherine Ward, World Bank

**Affiliations:** 1 The World Bank, Washington, DC, USA; 2 Global Health Academy/Usher Institute, Center for Medical Informatics, College of Medicine and Veterinary Medicine, Chair of Primary Care Research and Development/Deanery of Molecular, Genetic and Population Health Sciences, University of Edinburgh, Edinburgh, UK

## Abstract

While there is tremendous promise to leverage technology for UHC, it will require smart, context-specific policies and programming with ample flexibility to adapt as needs and opportunities change – and with robust safeguards to protect privacy, data security, and equity. The health sector, by its very nature of being data intensive, lends itself to the use of technology for analytics to improve health outcomes, respond to public health crises, and efficiently and equitably allocate resources. The first imperative in considering the use of digital health to expand UHC is to remember that digital health is a means to an end, and only one of the available means. Efforts leveraging digital health to move along that path to universality have taken many forms: to increase the number of people reached, to provide enhanced service coverage, and to reduce the financial burdens on individuals in need of health care. Making use of digital health interventions is an evolving process, not a one-time decision point. It is context specific and needs a clear vision to move from pilot interventions to scaled implementation. Technology can be a key tool in achieving UHC but its use has to be strategic, judicious, and cognizant of issues around privacy and patient rights.

## UNDERSTANDING UHC AND TECHNOLOGY

### What is Universal Health Coverage?

Universal health coverage is a central component of the Sustainable Development Goals (SDGs). Target 3.8 of SDG 3 states:

“Achieve universal health coverage, including financial risk protection access to quality essential health-care services and access to safe, effective, quality and affordable essential medicines and vaccines for all” [1].

Universal health coverage requires a fundamental shift in how we define and provide health care: moving from a disease-specific approach concentrated on counting the number of services provided to a people-centered approach. This approach must efficiently and equitably provide predictive, pre-emptive, personalized and participatory health care that enables people to live healthy, productive lives in which clients are active participants in their own care: empowered to better determine their own health outcomes and able to understand, control, protect, and leverage their own health information. Global health is about people and the systems needed to support people to live health lives. It is a systems and delivery problem, not just a disease problem. Medicines and other interventions to prevent and reverse diseases only have value if they reach the people who need them and do so in a way that ensures they are properly and sustainably used.

This entails substantial, concrete progress on three main fronts:

1. Providing all people with access to services;
2. Providing the full spectrum of essential, quality health services; and
3. Protecting people from overwhelming financial consequences of paying for health.

**Figure 1:**
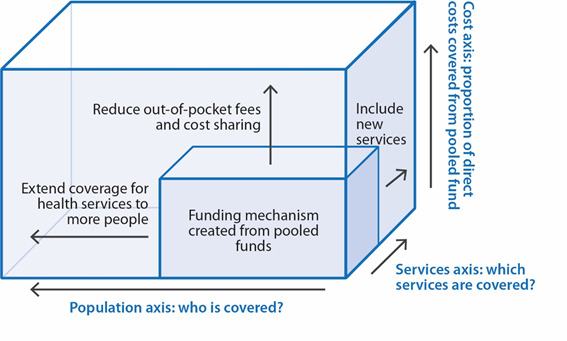
The Three Action Lines for Realizing UHC. Adapted from World Health Organization, by permission of World Health Organization [2].

In short, this means reaching more people, with more (and more effective) services, while also reducing the financial burdens on patients – a structure often explained using **Figure 1** [2].

### What does “technology in health” mean?

Technology. Big Data. Artificial Intelligence. Digital health. These are a few of the terms frequently used to describe this rapidly evolving set of tools that are often hailed as the answer to many health needs and challenges. But the first step to making use of them is to understand what they are and how they relate to one another. If we do not really know what the tools are, we cannot make smart decisions about the best ways to use them.

**Figure 2:**
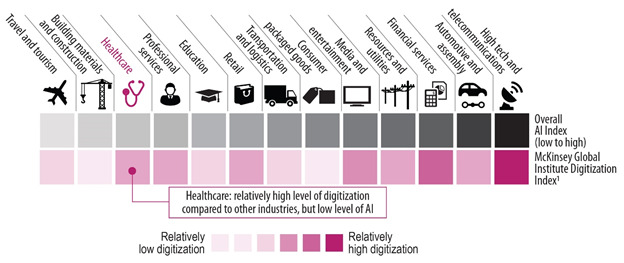
McKinsey Global Institute Digitization Index [7,8].

**Artificial Intelligence** (AI) is any task that would be considered intelligent if done by a human [3]. AI systems typically demonstrate behaviors associated with human intelligence such as planning, learning and reasoning. Its four core technologies are the three areas of **Big Data** analytics (computer vision and image recognition; natural language processing; and machine learning to cluster, predict, and classify) along with smart devices and robots. AI adapts through progressive learning algorithms to analyze more and deeper data, with increasing accuracy to get the most out of the health data we have. This is particularly relevant because health systems generate vast amounts of data. By 2020, the volume of health data was expected to exceed 2314 exabytes, with 2.5 trillion megabytes added daily; one report found that the expected compound annual growth rate of health care data between 2018 and 2025 will be 36 percent [4]. Currently, 30% of all global data are health data and 80% of health data are unstructured [5,6]. The McKinsey Global Institute Digitization Index has found that despite AI’s potential, uptake in the health sector is slow compared to other sectors, even in industrialized nations in Europe and the in the United States (**Figure 2**) [7,8].

AI can be used to answer a number of types of questions:

- What has happened? (descriptive)
- Why it happened? (diagnostic)
- What will happen? (predictive)
- What can we do? (prescriptive)

With machine learning analytics and thinking, AI can also be used to make recommendations about what to do based on the answers to those questions.

While AI has existed for decades, its potential has increased exponentially in recent years because of dramatic improvements in computational power, the rise of Big Data (rapid digitization and vast amounts of data now available, and the rise of fast and cheap data storage). The estimated amount of space necessary to store a million trillion megabytes has dropped from a data center equal to the size of Lithuania (2010 estimate) to the size of a tennis ball (2019 estimate). The rise of 5G networks will further facilitate data use, allowing such networks to move more data and move data faster. This will enable wider use of tools relying on videos, imaging, and continual patient monitoring using sensors [9]. One caveat that must be kept in mind is that AI is only as good as the data it uses. As a result, realizing the potential of AI also depends on making the requisite structural improvements in data collection.

Another common term is **digital health** which includes eHealth, mHealth, diagnostic innovations, the Internet of Things, and AI. In short, as defined by the World Health Organization in its 2019 Digital Health Guideline, digital health is the use of digital technologies, employing routine and innovative forms of information and communications technology to address health needs [10]. It is a more precise term than “technology” with a more clearly defined meaning and relevance for the topic at hand and will be used throughout the remainder of this article.

## USING DIGITAL HEALTH TO EXPAND EACH SIDE OF THE CUBE: APPLYING DIGITAL HEALTH TO UHC

The health sector is knowledge-intensive, dependent on data and analytics to improve health outcomes, respond to public health crises, and efficiently and equitably allocate resources [11]. The first imperative in considering the use of digital health to expand UHC is to remember that digital health is a means to an end, and only one of the available means. The focus should always first be on identifying the question that needs to be answered; only after that has been done should attention turn to determining how technology can be used to meet to answer that question [12]. It is also important to keep in mind that the focus should always remain on ensuring real benefits for real people in the health services value chain, with potential beneficiary groups including: individuals/patients; families; communities; clinicians/service providers; payers; regulators/policy-makers; and producers (eg, of medicines and equipment).

As indicated above, in the context of UHC the three overarching questions are:

- How to reach more people (risk stratification and targeting)? (benefits individuals, families, communities, clinicians and regulators);
- How to improve service quality (including new services)? (benefits individuals, families, communities, clinicians, payers, regulators and producers);
- How to strengthen financial risk protection (reducing financial burdens on patients while also ensuring a sustainable financing structure)? (benefits individuals, families, clinicians, payers, and regulators).

This entails finding smart, evidence-informed interventions for the following steps and determining what role, if any, digital interventions can and should play in those responses:

- Targeting the right areas (location services): finding those most in need and better locating services
- Making the right predictions (predictive analytics): identifying who will need help, and supporting patients as well as front-line and hospital-based health workers with remote diagnostic tools, monitoring, and consultative support
- Creating the right access (financial inclusion): eg, mobile payments/credits
- Ensuring the right recipients are reached (digital verification)
- Ensuring the right payments are made
- Providing the right job aids
- Making the right choices (data-driven choices)
- Delivering the right services
- Using the right provider (match need with expertise)
- Delivering the right value (technology solutions) (improve cost-effectiveness of health impact with available funds)
- Making the right measurement (performance innovation)
- Supporting the right innovation (accelerated R&D)

To this end, digital health, and particularly AI, can also help answer questions such as those presented in **Table 1**.

**Table 1:** Examples of questions digital health, and particularly AI, can help answer

	Who benefits?						
**Intervention example**	**Individuals (Patients)**	**Families**	**Communities**	**Clinicians**	**Payers**	**Regulators**	**Producers**
How do I proactively prevent disease from occurring?	×	×	×	×	×	×	
What other disease is this person likely to have that we should try to prevent?	×	×		×	×	×	
Which patients are more likely to acquire serious infections?	×			×	×		
Can we intervene sooner with treatments for patients who are at risk for dangerous complications?	×	×		×	×		
What is the likelihood that a patient will be readmitted after discharge?	×	×	×	×	×	×	
What is the likelihood that a person will miss their appointment?	×	×	×	×	×	×	
How many days will a patient need to stay in hospital?	×	×	×	×	×	×	
How many and what kinds of health workers will we need each night?	×	×	×	×	×	×	
How much money will a patient cost the health system over the next year?	×	×	×	×	×	×	×

These questions are clearly relevant to accelerating progress towards UHC. Digital health can also be used to improve health impacts in underserved settings by increasing capacity and efficiency by responsibly shifting tasks to individuals with less, but adequate, training (**task-shifting**); equipping individuals with the knowledge and tools to manage their own health (**self-management**); and using technology to address underlying risk factors, improve early detection, and identify cost-effective, high-impact measures and adjust resources accordingly (**improving population-level outcomes**) [10].

## EXPERIENCES AND INSIGHTS FROM THE FIELD

### Examples from the field

**Figure 3:**
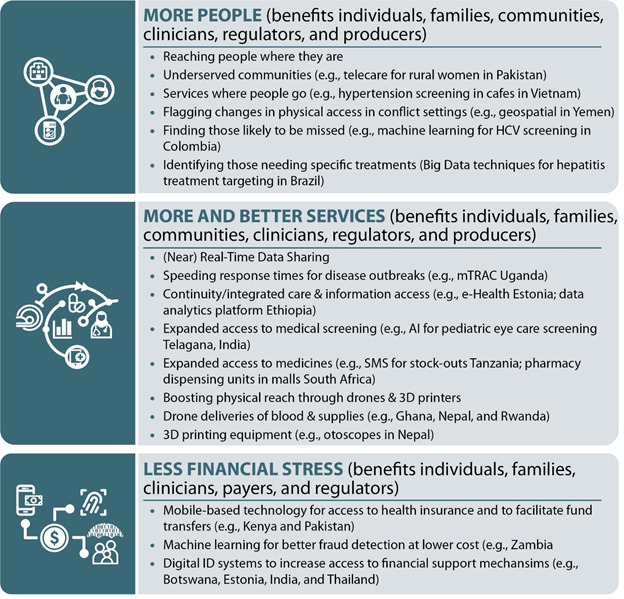
What helps and how it helps: examples from the field.

Digital health tools can be used in many ways to address a wide variety of health needs related to expanding UHC. These can include simple, single-tool interventions to achieve immediate, discreet successes. Or it can entail working over the longer-term to build integrated systems for broader impact. The bottom line is that using digital health to accelerate UHC is a process not an action point. Moreover, as some of the later examples below demonstrate, sustained success also requires attention to the building blocks that create a supportive environment for digital health. The following examples are organized according to the three action pillars of UHC described above: expanding access to more people, providing more (and better) services that meet people’s real health needs as part of a people-centered approach, and reducing financing burdens on individuals (reducing financial stress). It also includes a brief discussion of the area of digital identification, which often has cross-cutting impacts. It concludes with a few examples of more complex programs involving multiple forms and phases using digital health tools to provide insights into the complexities of more systematically integrating digital health into ongoing programming. **Figure 3** and **Table 2** summarize the interventions discussed and the groups that benefit from each.

**Table 2:** Digital health and UHC in action: examples of expanding the cube

	UHC cube side addressed			Who benefits?						
**Intervention example**	**More people**	**More & better services**	**Financial stress reduced**	**Individuals (Patients)**	**Families**	**Communities**	**Clinicians**	**Payers**	**Regulators**	**Producers**
Online telecare-enabled services for rural women in Pakistan	×			×	×	×	×		×	
Hypertension screening in cafes & nail salons in Vietnam	×			×	×			×		
Flagging changes in physical access to facilities during conflict (Yemen)	×			×	×	×			×	
Machine learning to identify HCV-risk individuals who would otherwise be missed in Colombia	×			×	×		×	×		
Big Data to reach individuals for specific hepatitis drug treatment in Brazil	×			×	×	×	×			×
Machine learning to identify high-risk for diabetes & other chronic diseases in Costa Rica	×			×	×		×	×	×	
SMS-based data reporting in Uganda tracking service quality, drug access & disease outbreak patterns		×		×	×		×	×	×	×
e-Health records & platform in Estonia		×		×	×		×	×	×	
Unified data analytics platform in Ethiopia		×		×	×	×	×	×	×	
AI for childhood eye care screening in India		×		×	×		×	×		
SMS system for drug stock-outs in Tanzania		×		×	×	×	×	×	×	×
Pharmacy dispensing units in malls with remote consultations in South Africa		×		×	×		×	×		×
Drones delivering blood & medical supplies in Ghana, Nepal & Rwanda		×		×	×	×	×			×
3D printing of otoscopes in Nepal		×		×		×	×		×	×
Mobile-based health insurance wallet facilitating access to health insurance & making payments, cash transfers easier in Kenya & Pakistan			×	×	×	×		×	×	
Machine learning to detect fraud more efficiently & at lower cost in Zambia			×	×				×	×	
Digital ID systems in Botswana, Estonia, India, South Korea & Thailand among others	×	×	×	×	×		×	×	×	
Suite of Big Data, GIS, & machine learning tools to refocus health system in Peru with changing disease burden	×	×	×	×	×	×	×		×	
Digital health program in Pakistan (Punjab), using multiple rounds & types of innovation (apps, GIS, machine learning) to boost vaccination & staff performance	×	×	×	×	×	×	×	×	×	

HCV – Hepatitis C virus

#### Reaching more people

Expanding access to care to more people is fundamental to any universal health coverage plan that aims to indeed be universal. Efforts leveraging digital health to move along that path to universality have taken many forms. The examples below illustrate some of the many promising paths that have been explored.

In Pakistan, to reach underserved communities with quality care, DoctHERS & Sehat Kehani use an app and web portal to provide online telecare, connecting over 40 000 patients in remote areas to qualified home-based female doctors who were not practicing [13]. Frontline health workers visit communities with app-enabled devices and also work in telemedicine centers, providing patients with two ways to tap into the systems, which now has over 20 eHealth telemedicine hubs and has almost 1 million beneficiaries [14,15].

In Colombia, a World Bank project is using machine learning to optimize resource management for the Hepatitis C virus (HCV) to better identify those likely to contract HCV but not be diagnosed and who will need a particular combination of medicines. Early results show machine learning can make these predictions with a high degree of confidence, although further progress has been slowed due to data access issues. A similar program in Brazil is using Big Data techniques to assess viral hepatitis treatment in the public health system. This project involves multi-step assessment integrating government databases, identifying the municipalities with the highest vulnerability for both detection and treatment of cases, and using machine learning to identify patients with vulnerabilities not covered in existing protocols who could benefit from screening.

In Vietnam, with support from PATH and Novartis, the government’s Communities for Healthy Hearts initiative in Hanoi works to reach people where they are with hypertension screening services provided in locations such as markets, nail salons, and coffee shops. Preliminary results indicate significant improvements in the number of people screened and the number of people receiving treatment, with 82% of people diagnosed with hypertension through the initiative currently under treatment, compared to 13.6% of hypertension patients managed at care facilities before the initiative began [9].

Overstretched health care facilities in China have led officials to promote greater use of telemedicine. The resulting programs include Ping An Good Doctor, an online platform established in 2015 to provide online consultations and appointment bookings. Within two years, it had almost 200 million registered users and over 9000 doctors serving those users [9].

To identify and reach high-risk patients in Estonia, the World Bank is also conducting an innovative data analysis that uses data analytics and incorporates medical diagnoses and socio-economic data on individuals to better identify patients who were harder to find using traditional economic prediction methods. In Costa Rica, it is using machine learning algorithms to identify patients at high risk of developing diabetes and other chronic health issues such as hypertension, so health brigades can use the risk profiles to take preventive action. In Yemen, it has been able to use GIS to estimate access to health facilities and how that access has changed over time—an example of a situation in which digital health interventions may be the only option as conflict precludes data collection through more traditional methods such as census taking.

#### Providing more (and better) services

Examples of health interventions leveraging digital health to provide enhanced services cover a myriad of needs employing a wide variety of types of digital health tools, of which the following is only a small sample.

Near real-time data sharing through mTRAC, an SMS-based health reporting program in Uganda to track service quality, drug access, and disease patterns in Uganda, is being used by over 53 000 health workers and was credited with halving response time to disease outbreaks. In 2015, it allowed the ministry of health and district response teams to respond to a typhoid outbreak within hours of its emergence [16].

AxisMed Brazil has successfully used personal devices provided to chronic patients to track and transmit biometric data to help medical professionals better oversee their treatment plans. Under the intervention, over 80% of the monitored patients have adhered to their treatment plans and emergency department visits have reduced by two-thirds [9,11,17].

The e-Health records system and portal in Estonia ensures that every person in the country has an online medical history and integrates data from across health care providers into a single record for each patient. The patient can use it to track their care and receive general health advice, while health care providers can access records to better understand the totality of their patient’s health profile. The electronic health record system pools health data from multiple sources into a single record for each patient using X-Road, a central information exchange layer, that uses blockchain technology to protect data privacy and integrity [9,11].

In Ethiopia, in just 12 months, the Federal Ministry of Health (FMOH), with support from Zenysis, integrated health data from more than 10 disconnected systems into a single platform: the Ethiopian Data Analytics Platform. The FMOH then used the platform to optimize its immunization program and to conduct data analytics that led it to allocate over $100 million to maternal and child health programs [9].

In the Indian state of Telagana, the government adopted Microsoft Cloud and became the first state to use AI for eye care screening for children [18]. And in Tanzania, an SMS system to report drug stock-outs to a GIS-enabled command center was able to rapidly halve drug stock outs [19].

In a practice that could be a game-changer for managing chronic diseases, the Right to Care group in South Africa has installed 5 pharmacy dispensing units in shopping malls and hospitals in Johannesburg. The units connect users to pharmacy assistance to remote consultations before dispensing prescribed medicines — in a process that takes only a few minutes in total. In a country that provides free treatment to 4.2 million people living with HIV and regularly sees people waiting hours to have their prescriptions filled, this offers tremendous promise to slash waiting times at clinics, relieving pressure on patients and the health care system [20,21].

Drones and 3D printers have also been explored as ways to boost physical reach where limitations in the transportation infrastructure pose barriers to effective access to critical services and supplies. To improve access to blood and medical supplies at hospitals in the southern and western sections of the country, the government of Rwanda has successfully partnered with Zipline, a California-based robotics firm, to deliver these items, reducing the delivery time from four hours or more to about 30 minutes [22,23]. More recently expanded to Ghana as well, Zipline’s work in the two countries now delivers over 170 different vaccines, blood products, and medications to 25000 health facilities in the two countries, serving 22 million people [24]. This example also embodies the concept of leapfrogging, where countries without well-established pre-existing technologies can use emerging technologies to meet a concrete health need without having to invest in the previous, and often more expensive technology (in this case road improvements) [9]. Inspired by the Zipline example, Nepal’s National Innovation Centre is piloting the Medical Drone Project to deliver medicine packages and laboratory samples to and from remote health posts; and the humanitarian NGO Field Ready deployed a 3D printer in the village of Bhotecaur to print otoscopes on site and at lower cost for immediate use when poor roads, financial constraints and slow bureaucracy made it difficult to access these basic devices in remote facilities [25,26].

Big Data analytics show promise as well. For example, in South Africa, the National Health Laboratory Service and National Department of Health, with support from the World Bank and Boston University, used Big Data analytics to boost HIV service coverage and quality by identifying and targeting support to underperforming facilities [27].

Finally, health technology assessments can provide decision-makers with quality information to help determine how best to spend available funds—effectively increasing the services available without budget increases. For example, Thailand was an early pioneer among its peers in using of insights from HTAs to develop its universal coverage benefit package. More recently, Turkey formalized HTAs for hospital services to bolster affordability and efficiency [27].

#### Reducing the financial burden on individuals (out-of-pocket expenses and cost-sharing)

Reducing the financial burdens on individuals in need of health care is the critical third pillar in the architecture of universal health coverage. In addition to the programs described below, digital health tools such as data analytics and data-driven efficiency assessment modelling tools can be used to improve service targeting and reduce waste. This can reduce some service delivery costs and free up additional money in existing budgets which in turn can be used to create mechanisms to further reduce financial barriers to access.

*Connecting people to health financing.* In Kenya, 40% of Kenyans who need care cannot afford treatment, nearly 50% of health care expenditures are out-of-pocket expenses, and the traditional, paper-based insurance and voucher systems to help people afford care are slow, unreliable, and not trusted. M-Tiba provides a mobile-based health insurance wallet (Kenya) that users can access to send, save, and spend funds to pay instantly through M-Pesa for medical treatment at over 350 facilities. The health wallet can be used for health savings, health insurance coverage funds and for free health benefits provided by the government or donors, giving those in need immediate, secure access to funds. The program has almost 1 million users, with over 4000 new users per day and has made over US$1 million in medical pay-outs since its launch in 2016 [28]. The National Hospital Insurance Fund has also partnered with M-Tiba to provide 2000 households with health care insurance and CarePay and Safaricom activated “send money” features allowing people to send health care funds from their M-Pesa accounts to the M-Tiba accounts of others including relatives and household staff [29,30]. The underlying M-Pesa system reaches 93% of Kenyans. In Pakistan, Easypaisa, launched in 2009, is now the world’s third-largest mobile bank, serving over 18 million people [31]. Keys to success for the Kenya and Pakistan programs have included: streamlined enrolment, governments use the service to disburse payments to individuals, and reduced regulatory requirements by regulating the service as a money transfer service rather than a bank—thus making access easier for the unbanked who are unable to access traditional banking because of regulatory requirements [32-34].

*Increasing impact of available health financing support funds for individuals by decreasing fraud.* In Zambia, a World Bank team has shown that machine learning approaches, particularly Random Forest, outperformed other fraud detection methods and increased the cost-effectiveness of verification [35].

#### Digital identification for health

As countries move toward universal coverage, many new insurance schemes are emerging. Integrating an ID system into these schemes can increase efficiencies and expand inclusion [11]. These ID programs can help “make the invisible visible” providing identification to those who lack it so they can access social benefits including health care, which, in turn, can reduce the financial burdens they face. To this end, the World Bank Group’s Identification for Development (ID4D) initiative, with support from the Bill & Melinda Gates Foundation, the Omidyar Network and the Australian Government, launched the Mission Billion Challenge in November 2018, to spur efforts to provide IDs to the estimated one billion people globally who currently lack them [36].

In the context of UHC, digital identification systems have been shown to produce benefits across the three main pillars of action to achieve UHC described above, fostering gains in patient management and treatment, insurance and benefits programs, and data collection for planning and population-level health improvement. Foundational ID systems have received particular attention in this regard. In Estonia, the digital ID card, which is mandatory for all citizens and legal residents over 15 years of age, and the automatic registration at birth of all citizens in the Population Register have been linked to the Estonian Health Information System to expand health care coverage. This includes guaranteeing all children are covered by the national compulsory health insurance program from birth [11]. In Botswana, linking the national Omang ID card system to the country’s flagship antiretroviral therapy program and related services for diseases such as tuberculosis has given patients easier access to needed support [11].

In Thailand, the BORA identification system, established in 1984, has been integral in operationalizing the country’s Universal Coverage Scheme (UCS), which guarantees subsidized health care to all citizens. The national ID registers all Thai citizens, eligible migrants, refugees, and stateless people and gives them a unique personal identification number (PIC). Leveraging this system, the UCS reportedly reduced the uninsured portion of the population from 29% to under 5% in under two years [9]. It also ensures that individuals are automatically moved from one portion of the insurance rolls to another when changes in their work status change the way they are covered. Similarly, in South Korea, the National ID card and ID numbers are integrated into the health care system, supporting the National Health Insurance program, which provides universal health care services, covering close to 97% of the population [11,37]. And technologies from India’s Aadhaar national ID number system, which had issued over 1.19 billion unique ID numbers by the end of 2017, are being used to track the performance of health workers and improve the process of identifying insurance beneficiaries [9,11].

#### Complex interventions: combining multiple digital health tools in multi-phased responses tied to an overarching goal

In the longer run to more fully leverage the power of digital health requires more complex interventions. These multistage processes often entail building from small, discreet interventions, learning from what did not work, committing to flexible persistence, and integrating the digital health components with many other components that are essential to producing the desired health outcomes. The following two examples offer quick views of different experiences in this regard.

Addressing a need can also entail using multiple types of digital health tools. For example, a World Bank team in Peru is using Big Data (data analytics), GIS, and machine learning to support government decision making to refocus the health system, starting in Lima Province, in light of changing disease burdens. Still in its early stages, the Health Networks Transformation initiative is charged with improving the health of those who lack access to health services, while also improving the governance, efficiency, and equity of the health system: addressing growing needs related to chronic conditions and NCDs, reducing service fragmentation across small facilities, and creating referral pathways to ensure people receive the care they need. In a three-component, integrated initiative, the team has already used large-scale HIS data and analytics to produce a first-round of a strategic map to optimize the network facilities based on current usage and gaps and predictions of future demand. It has also been able to produce preliminary assessments using GIS tools (eg, OpenStreetMap and Waze) and local health data to determine the optimal locations for health facilities to maximize services to meet identified health needs while minimizing costs. Finally, it is using Big Data and machine learning techniques to improve the targeting of care by using machine learning to identify high-performing health facilities and their attributes. While early results are promising, they are preliminary. They have also required the use of a gradual, iterative approach to allow for consensus and trust building, to approach data challenges in terms of both access and quality, and to allow decision-makers to set benchmarks for what the initiative should achieve and how to prioritize various service goals.

As the case study from Pakistan in **Box 1** demonstrates, moving from reactive interventions addressing an immediate need to a more systematic program that more fully leverages the potential of digital health to extend and improve health services and outcomes is an iterative process requiring perseverance and attention to a number of other factors beyond the digital health tools.

Box 1 **Phased progress towards a systemized use of digital health: multiple rounds of innovation and multiple keys to success – a case study from Punjab**

The widely lauded digital health program in the Punjab region of Pakistan did not start out as a grand plan. Its origins lay largely in an unsuccessful attempt to counteract a dengue outbreak in 2011. It began as a straightforward effort using a single intervention to address a single challenge is a small area: providing health inspectors with smartphones loaded with a special app to use in inspecting health care facilities in a single district. The plan came about too late to succeed. However, with leadership from Shehbaz Sharif, the chief minister of Punjab, the failure became a building block to create a plan for future success: leading to the development of a system, leveraging World Bank technical assistance and financing from its Innovation Fund, in advance of the 2012 dengue season to monitor prevention activities and identify hotspots across Punjab. With funding secured, the government expanded its small, existing IT department to hire software developers and other technical staff including highly capable computer scientists and a manager to develop custom-tailored apps, and partnered with a local university to develop Data Plug, a specialized support platform. The resulting apps were then loaded onto smartphones and distributed to inspectors; and the program was credited with helping to slow the spread of dengue in 2012 in the region. Then, building on success and innovating again for broader impact, further collaborative innovation between the provincial government and the World Bank explored further potential gains, conducting a randomized control trial that expanded the project to half of Punjab’s districts and showed a large increase in attendance at facilities that were monitored by smartphone-equipped inspectors.

Insights from the trial were used to build again: this time to develop a broader Punjab Management Reform Program, launched in November 2013 targeting five departments including health. A new app for vaccinators distributed on smartphones in 2014 required vaccinators to use the app to check in and upload data at several designated locations during the work day. Vaccinator attendance increased from 36% to over 80% in just four months. But the locations covered remained static, focused on places easiest for the vaccinators to reach. This led to another round of technical partnership and innovation to produce a mapping system (using GIS and machine learning) that divided the province into polygons representing each neighborhood and provided real-time reporting to indicate when a neighborhood was not receiving enough visits so health officials could quickly redeploy vaccinators to rectify the problem. Result: the percentage of polygons reached by vaccinators rose from 25% when the program was launched in 2014 to 88% by May 2016 and the percentage of fully immunized children rose from 62% to 81%, with 95% of children fully vaccinated against polio. By 2017, health inspectors were using the updated application and a counterpart app to monitor over 3000 facilities across the province to check staff attendance, medicine availability, equipment conditions, and facility maintenance, with the uploaded information linked to a dashboard the health department could use to track the performance of both hospitals and inspectors. Pressure on inspectors to inflate results also decreased since reporting became instantaneous and automated, and transparency improved with the Punjab government creating an open government website (http://open.punjab.gov.pk) that made data from multiple departments and projects including the vaccination program available to the public.

Lessons learned: getting the technology right is critical but not enough. Success also depended on: (i) building positive relationships between IT teams and other staff and users so they could collaborate to “co-create” solutions; (ii) political leadership; (iii) increased institutional capacity; (iv) providing incentives for people to use the intervention; and (v) creating transparency [38,39].

### Insights from the field: key principles learned

The following insights, gained from experience implementing digital health interventions to date, provide learned key principles that should be leveraged as we strive to move towards smarter, systems-oriented decision-making on how to use and integrate digital interventions into planning and programming to achieve UHC.

#### From low-hanging fruit to moonshots

As seen in the Punjab example, making use of digital health interventions is an evolving process, not a one-time decision point. As needs and capabilities evolve, so do the options for effective use of digital interventions. Uses that would have been impossible at first, become attainable as capabilities improve, allowing decision makers-to move from using simple interventions to solve easy “low-hanging fruit” challenges to eventually taking on larger “moon-shot” challenges such as a fully integrated, eHealth system to manage universal health care as is under way in countries such as South Korea, Thailand, and Estonia.

This also ties into another important key need going forward: a sustained press to ensure movement from piloting to systematic reform. Using data to reshape health systems can and should be a key pillar of this effort.

#### Distinguishing between hype and substance: informed technology selection

To date, health decision-makers have lacked resources they can turn to for evidence-based methods that can be used to determine which digital health intervention (if any) is well-suited to meet a particular need in their situation. Efforts are under way to help countries better tackle these challenges, although much more work remains to be done. The World Health Organization’s 2019 Guideline *Recommendations on Digital Interventions for Health System Strengthening* is the first such guideline by the organization and is designed to help decision-makers make informed decisions about which digital health interventions to invest in [10]. The US Agency for International Development’s Center for Innovation and Impact is also partnering with the Bill & Melinda Gates Foundation and The Rockefeller foundation to explore use of AI in global health and to share its findings with stakeholders around the globe through various means including the report *Artificial Intelligence in Global Health* [40]. The long-term goal must be to create reliable, evidence-based methods for such decision-making that can be adapted to take into consideration the needs, priorities, strengths, and limitations faced in a particular context at a particular time.

#### Moving beyond a use-case mentality: creating AI-enabled health delivery systems and analytics

At first, a health system’s use of digital health interventions is often episodic: identifying a particular tool that can address a discreet, immediate need within a particular health intervention area, such as a smartphone app to share HIV prevention messages with at-risk youth in a particular area or community. However, to fully leverage the potential of digital interventions to further the goal of people-centered, sustainable, and equitable UHC, use of digital interventions should, as capabilities develop, shift to developing broader delivery systems and analytics that are fully AI-enabled. Developments in South Korea and Estonia provide examples of varying paths towards this goal.

#### Identify and be clear about likelihood of improved impact and the likely amount of gain

Determining that a particular digital intervention would have an impact is not enough. Those conducting such assessments should be careful to make sure their evaluations reflect the effects of size and variance. This is a widespread issue, but particularly true of “nudge” interventions that would have a marginal impact on outcomes. It is also important to note that analysts too often tell decision-makers that *x* “works” rather than stating that doing *x* may improve outcomes fractionally.

#### Better understanding v. better action: bridging the gap

There are still many cases in which there is a failure to translate insights about what is happening and why it is happening into concrete policies and programs that actually improve health outcomes.

#### Digital context

Digital health interventions should only be used when they have been properly vetted and determined to be well suited to addressing a given need. But even in those conditions, they will be hard pressed to succeed unless the necessary support environment exists. For example, as noted in the report of the Working Group on Digital Health of the Broadband Commission for Sustainable Development, *The Promise of Digital Health: Addressing Non-communicable Diseases to Accelerate Universal Health Coverage in LMICs*, to maximize impact, such interventions must be supported by foundational building blocks including: vision and leadership, regulations and policies, communications infrastructure and common platforms, interoperability frameworks (to allow different data sources and systems to connect with each other), partnerships involving a variety of stakeholders, and sustainable financing models [9].

#### Country context

As the examples above demonstrate, countries have different needs, different strengths, different priorities, and different limitations. In addition, as seen in the multi-year, multi-phase project in Pakistan/Punjab, those factors can also change for a given community over time. To succeed, digital health interventions must reflect and adapt to those realities. Efforts to develop stakeholder understanding, trust, and inclusion are also critical as are effective measures to address social and economic inclusion needs.

#### Data: access, quantity, quality and ability to share

The existence of sufficient, relevant, reliable data should not be taken for granted, even in countries that have made significant progress in establishing eHealth records. Moreover, even when quality data exist and privacy standards have been protected, the data owners may want to establish a sense of trust in the proposed intervention plan before agreeing to share the data.

#### Privacy and control of data

As touched upon above, privacy is and should be a primary concern in establishing programs and processes that protect individuals’ rights to privacy and enable them to have greater control over the use of their data. Legal and regulatory frameworks are a critical component of effective systems to protect data privacy. Much work remains to be done, but some progress is being made. For example, the 2014 African Union Convention on Cyber-Security and Personal Data Protection sets standards that can be used to both establish domestic frameworks and to harmonize those frameworks to strengthen data process across the region [9]. The World Bank’s Principles on Identification for Sustainable Development also offer guidance on data protection, security, and privacy in contexts involving unique IDs and health systems [41].

Blockchain technology may prove useful in addressing certain types of privacy concerns. For example, Estonia uses blockchain to ensure data privacy and integrity in its national e-Health system. Furthermore, as noted in a report by the Broadband Commission, blockchain may be particularly useful in safeguarding health data privacy in UHC settings, which, by definition, entail connecting large amounts of health data from different sources. That said, use of blockchain in health is still generally in early stages of development because the legal and regulatory framework, technical capacities, and incentive structures are still under development. In the interim, the use of personal identification codes in countries with digital health IDs such as Thailand and Estonia has helped to reduce some of the risk [9].

While these insights and examples from the field do not provide a perfect roadmap for every country working to leverage technology to achieve UHC, they do provide important road signs to help countries to stay on course and avoid numerous detours that would slow progress. It is also important to remember that success ultimately also depends on creating a supportive enabling environment including the legal, logistical, and infrastructure frameworks on which digital health tools and UHC depend. However, as noted in this chapter, the promise is real and substantial. And given the scale of the challenge of achieving effective and robust UHC for all people in all countries—a challenge which the COVID-19 pandemic has both heightened and highlighted—these opportunities must be seized and realized.

## References

[1] United Nations. Sustainable Development Goal 3, Target 3.8. Available: https://sustainabledevelopment.un.org/sdg3.

[2] World Health Organization. Arguing For Universal Health Coverage. Geneva: World Health Organization; 2013. Available: https://www.who.int/health_financing/UHC_ENvs_BD.PDF. Accessed:1-Nov-2019.

[3] Minsky M. Information Processing. Cambridge: MIT Press; 1968.

[4] Reinsel D, Gantz J, Rydning J. Data Age 2025: The Digitization of the World From Edge to Core. International Data Corporation (IDC). 2018.

[5] Frye E, Mukherjee S. Tech’s Next Big Wave: Big Data Meets Biology. Fortune. 2018. Available: https://fortune.com/2018/03/19/big-data-digital-health-tech/. Accessed: 1 August 2021.

[6] Kong H-J Managing Unstructured Big Data in Healthcare System. Healthc Inform Res. 2019;25:1-2. 10.4258/hir.2019.25.1.1 30788175 PMC6372467

[7] McKinsey Global Institute. Digital Europe: Pushing the Frontier, Capturing the Benefits. McKinsey Global Institute. 2016.

[8] McKinsey Global Institute. Digital America: A Tale of the Haves and Have-Mores. McKinsey Global Institute. 2015.

[9] Broadband Commission. The Promise of Digital Health: Addressing Non-Communicable Diseases to Accelerate Universal Health Converage in LMICs. Broadban Commission for Sustainable Development. 2018.

[10] World Health Organization. WHO Guideline: Recommendations on Digital Interventions for Health System Strengthening. Geneva: World Health Organization; 2019. 31162915

[11] World Bank. The Role of Digital Identification for Healthcare: The Emerging Use Cases. Washington, DC: World Bank; 2018.

[12] Goldberg PK. Data at the World Bank: Moving Beyond the Hype. Washington, DC: World Bank Group; 2019.

[13] Bhattacharya A. Pressured to Give Up Their Careers, Pakistan's “Doctor-Wives” are Using Tech to Find Work Again. 2017. Available: https://qz.com/india/1064758/sehat-kahani-pressured-to-give-up-their-careers-pakistans-doctor-wives-are-using-tech-to-find-work-again/. Accessed:1 November 2019.

[14] Kahani S. What is Sehat Kahani? Available: https://sehatkahani.com. Accessed:1 November 2019.

[15] Middleton J, Ajadi S, Bayen M. Ecosystem Accelerator Compass: Insight on Start-ups and Mobile in Emerging Markets. GSM Association. 2019.

[16] SDSN TReNDS. Data Sharing via SMS Strengthens Uganda's Health System: A Case Study of mTRAC, Uganda. Available: https://www.data4sdgs.org/sites/default/files/2018-09/mTRAC%20CaseStudy_FINAL.pdf. Accessed:4 November 2019.

[17] World Bank. Estonia: A Successfully Integrated Population-Registration and Identity Management System: Delivering Public Services Effectively. Washington, DC: World Bank; 2015.

[18] Microsoft News Center India. Government of Telangana Adopts Microsoft Cloud and Becomes the First State to Use Artificial Intelligence for Eye Care Screening for Children. 2017. Available: https://news.microsoft.com/en-in/government-telangana-adopts-microsoft-cloud-becomes-first-state-use-artificial-intelligence-eye-care-screening-children/. Accessed:24 October 2019.

[19] Barrington J, Wereko-Brobby O, Ziegler R. SMS for Life: Tanzania Pilot Project Report. Geneva: Roll Back Malaria; 2010.

[20] Right to Care. Medication ATMs Launched in SA: Patient Wating Times Cut to Under 3 Minutes. 2018. Available: https://www.righttocare.org/press-releases/medication-atms-launched-in-sa-patient-waiting-times-cut-to-under-3-minutes/. Accessed:1 November 2019.

[21] Mkhize D. ATM Pharmacy to Cut Queues for South Africa's AIDS Patients. 2018. Available: https://www.reuters.com/article/us-safrica-aids-arv-atm-idUSKCN1GR2CO. Accessed:1 November 2019.

[22] Marquez PV. Drones and Blood Safety Can Save Lives. 2019. Available: https://blogs.worldbank.org/health/drones-and-blood-safety-can-save-lives. Accessed:24 October 2019.

[23] Mwai C. Replicate Drones for Medical Supplies – World Bank President. New Times. 2017. Available: https://www.newtimes.co.rw/section/read/209327. Accessed 1 August 2021.

[24] De Leon R. Zipline Takes Flight in Ghana, Making it the World's Largest Drone-Delivery Network. 2019. Available: https://www.cnbc.com/2019/04/24/with-ghana-expansion-ziplines-medical-drones-now-reach-22m-people.html. Accessed:1 November 2019.

[25] Saunders S. Field Ready 3D Printing Much-Needed Medical Supplies for a Nepal Health Clinic. 2017. Available: https://3dprint.com/166818/3d-printed-otoscope-nepal-clinic/. Accessed:1 November 2019.

[26] Drupa. Humanitarian Aid through 3D Printing. 2019. Available: https://blog.drupa.com/en/humanitarian-aid-through-3d-printing-2/. Accessed:1 November2019.

[27] World Bank. Business Unusual: Accelerating Progress Towards Universal Health Coverage. Washington, DC: World Bank; 2018.

[28] Shared Value Africa Initiative. M-TIBA: Transforming Lives. 2018. Available: https://www.svai.africa/2018/10/03/m-tiba-transforming-lives/. Accessed:4 November 2019.

[29] Sturman C. The M-Tiba App is Revolutionising Healthcase in Kenya. 2018. Available: https://www.healthcareglobal.com/technology/m-tiba-app-revolutionising-healthcare-kenya. Accessed:24 October 2019.

[30] Macharia K. Health e-Wallet M-Tiba Signs up 60 Clinics in Nairobi. 2016. Available: https://www.capitalfm.co.ke/business/2016/09/health-e-wallet-m-tiba-signs-60-clinics-nairobi/. Accessed:24 October 2019.

[31] Howling Pixel. Telenor Pakistan. Available: https://howlingpixel.com/i-en/Telenor_Pakistan#cite_note-14. Accessed:4 November 2019.

[32] Burki H-B. Easypasia: Overview of the Operation of Mobile Money Service Provision. Grameen Foundation. 2013.

[33] Munda C. Transactions through Mobile Money Platforms Close to Half GDP. Daily Nation. 2017.

[34] Group RFI. Kenya 9 in 10 Kenyans are Financially Included Largely Thanks to M Pesa. 2016. Available: https://www.rfigroup.com/global-retail-banker/news/kenya-9-10-kenyans-are-financially-included-largely-thanks-m-pesa. Accessed:4 November 2019.

[35] Grover D, Bauhoff S, Friedman J. Using Supervised Learning to Select Audit Targets in Performance-Based Financing in Health: An Example from Zambia. Washington, DC: Center for Global Development; 2018. 10.1371/journal.pone.0211262 PMC6350980 30695057

[36] W. B. ID4D. Mission Billion. Available: https://id4d.worldbank.org/missionbillion. Accessed:24 October 2019.

[37] MOHW. KIHASA. Social Security Factbook 2016. Seoul: Korea Social Security Committee, Ministry of Health and Welfare. 2017.

[38] Martin J. Hospital-Based Technology Assessment: The Next Frontier. Hospital-Based Technology Assessment. Springer International Publishing. 2016.

[39] Beschel RP, Cameron BJ, Kunicova J, Myers CB. Improving Public Sector Performance through Innovation and Inter-Agency Coordination (Case Study 13 Using Smartphones to Improve Service Delivery in Punjab, Pakistan). Washington, DC: World Bank; 2018.

[40] United States Agency for International Development Center for Innovation and Impact. Artificial Intelligence in Global Health: Defining a Collective Path Forward. Washington, DC: United States Agency for International Development; 2019.

[41] World Bank. Principles on Identification for Sustainable Development: Toward the Digital Age. Washington, DC: World Bank Group; 2018.

